# Prenatal Administration of Betamethasone Causes Changes in the T Cell Receptor Repertoire Influencing Development of Autoimmunity

**DOI:** 10.3389/fimmu.2017.01505

**Published:** 2017-11-13

**Authors:** Anna Gieras, Christina Gehbauer, David Perna-Barrull, Jan Broder Engler, Ines Diepenbruck, Laura Glau, Simon A. Joosse, Nora Kersten, Stefanie Klinge, Hans-Willi Mittrücker, Manuel A. Friese, Marta Vives-Pi, Eva Tolosa

**Affiliations:** ^1^Department of Immunology, University Medical Center Hamburg-Eppendorf, Hamburg, Germany; ^2^Immunology Division, Germans Trias i Pujol Research Institute and Hospital, Universitat Autonoma de Barcelona, Badalona, Spain; ^3^Institute of Neuroimmunology and Multiple Sclerosis, Center for Molecular Neurobiology Hamburg, Hamburg, Germany; ^4^Department of Tumor Biology, University Medical Center Hamburg-Eppendorf, Hamburg, Germany; ^5^Centro de Investigación Biomédica en Red de Diabetes y Enfermedades Metabolicas Asociadas (CIBERDEM), Instituto de Salud Carlos III, Madrid, Spain

**Keywords:** glucocorticoids, prenatal betamethasone, T cell repertoire, autoimmunity, type 1 diabetes, non-obese diabetic mice, experimental autoimmune encephalomyelitis mice, MRL/lpr

## Abstract

Prenatal glucocorticoids are routinely administered to pregnant women at risk of preterm delivery in order to improve survival of the newborn. However, in half of the cases, birth occurs outside the beneficial period for lung development. Glucocorticoids are potent immune modulators and cause apoptotic death of immature T cells, and we have previously shown that prenatal betamethasone treatment at doses eliciting lung maturation induce profound thymocyte apoptosis in the offspring. Here, we asked if there are long-term consequences on the offspring’s immunity after this treatment. In the non-obese diabetic mouse model, prenatal betamethasone clearly decreased the frequency of pathogenic T cells and the incidence of type 1 diabetes (T1D). In contrast, in the lupus-prone MRL/lpr strain, prenatal glucocorticoids induced changes in the T cell repertoire that resulted in more autoreactive cells. Even though glucocorticoids transiently enhanced regulatory T cell (Treg) development, these cells did not have a protective effect in a model for multiple sclerosis which relies on a limited repertoire of pathogenic T cells for disease induction that were not affected by prenatal betamethasone. We conclude that prenatal steroid treatment, by inducing changes in the T cell receptor repertoire, has unforeseeable consequences on development of autoimmune disease. Our data should encourage further research to fully understand the consequences of this widely used treatment.

## Introduction

Non communicable diseases (NCDs), such as cardiovascular diseases, diabetes, chronic respiratory diseases, and others, result from a combination of risk factors and are the leading cause for almost 70% of all deaths worldwide (1). Among these, autoimmune diseases are responsible for more than 5% deaths every year (2), and their incidence is steadily increasing (3, 4). Emerging evidence suggests that prenatal and early life conditions, such as environmental exposure to pollutants, poor nutrition, stress, or medical interventions during pregnancy might act as pivotal determinants of NCD risk in later life (5). Fetal exposure to synthetic glucocorticoids, as consequence of antenatal corticosteroid (ACS) administration to the mother, constitutes a potentially amendable risk factor. ACS is the most important obstetric intervention available to reduce the occurrence and severity of respiratory distress syndrome and to improve the survival chances in premature infants (6). According to WHO recommendations, glucocorticoids (either betamethasone or dexamethasone) are given to mothers at risk of preterm birth between 24 and 34 weeks of gestation (7). The synthetic glucocorticoids cross the placenta and accelerate fetal lung maturation, achieving maximum benefit from 24 h to 7 days after administration (8). After injection, glucocorticoid bioactivity in the fetus lasts for several days (9) and can exert long-lasting effects upon the hypothalamic–pituitary–adrenal (HPA) axis and cognition in children (10, 11). Of note, 30–80% of women with symptoms of preterm birth have not delivered 14 days later (12), and more than half of the women who received ACS delivered later than one week after treatment or after 34 weeks of gestation (13), among them one third after gestation week 37 (14). Especially this population of neonates is likely to encounter more harm than benefits from the prenatal treatment with glucocorticoids.

Known as strong immune modulators, glucocorticoids act on a great variety of immune cells and have potent effects on the development of T lymphocytes. Exposure of developing thymocytes to glucocorticoids results in apoptotic death of the immature CD4^+^CD8^+^ double-positive (DP) subset (15), and we have previously shown that antenatal betamethasone treatment leads to a drastic decrease in the thymus volume and thymocyte numbers in the offspring (16). In order to sustain T cell output, the thymic niche is promptly replenished by newly imported hematogenous early thymic progenitors (17, 18), which will then proceed to the rearrangement of their T cell receptor (TCR) creating a highly diverse TCR repertoire and undergo positive and negative selection processes: thymocytes bearing non-functional TCRs or TCR with high affinity for self-peptide:MHC are deleted, while T cells with lower affinity TCRs mature and populate the periphery [reviewed in Ref. (19)]. However, even in healthy individuals, a number of autoreactive T cells escape this central tolerance checkpoint and migrate to the periphery, where peripheral tolerance mechanisms are engaged to prevent overt autoimmunity. Among these, thymic-derived Foxp3^+^ Treg cells and, in particular, a highly efficient population of Treg cells generated perinatally, are essential in preventing multiorgan autoimmune disease (20–23).

The delicate process of T cell repertoire selection may be compromised under altered glucocorticoid signaling (24–29), and as a consequence may have detrimental long-lasting effects on immunopathology and susceptibility to diseases. Therefore, we sought to investigate the influence of ACS treatment on the development of autoimmunity later in life. Using two mouse strains that spontaneously develop autoimmunity, namely the lupus-prone MRL/lpr strain and the non-obese diabetic (NOD) mouse, and the myelin oligodendrocyte glycoprotein (MOG)-induced mouse model for multiple sclerosis [experimental autoimmune encephalomyelitis (EAE)], we provide evidence that prenatal betamethasone treatment, by inducing changes in the T cell repertoire, can alter the course of autoimmune disease.

## Materials and Methods

### Mice and Treatment

C57BL/6J, MRL/MpJ-*Fas^lpr^*/J, NOD mice (The Jackson Laboratory, Bar Harbor, ME, USA), and Foxp3^RFP^ reporter mice (30), kindly provided by S. Huber, were housed and maintained under specific pathogen-free conditions. Mice were mated and the presence of a vaginal plug was considered day 0.5 of pregnancy (E0.5). On day 18.5 (E18.5) mice were treated by i.p. injection of 0.1 mg betamethasone (Sigma-Aldrich, Germany) in PBS or vehicle control (PBS). When indicated, 4- to 5-week-old animals were given 0.1 mg betamethasone i.p. or vehicle control (PBS) 24 h prior to organ harvesting. All animal experiments were performed in accordance with national and institutional guidelines on animal care and ethics.

### Flow Cytometry

Thymi, lymph nodes and spleens were harvested and single-cell suspensions prepared by mechanical disruption and filtering through a 70 µm nylon mesh. Red blood cells were lysed when necessary and Fc receptors were blocked using anti-CD16/32 Abs prior to staining. Anti-mouse Abs used in this study were: anti-CD3e eFluor 450 (500A2), anti-CD4 APC-eFluor 780 (RM4-5) and anti-CD8α PE-Cy7 (53-6.7) from eBioscience (San Diego, CA, USA); anti-CD4 FITC (RM4-5), anti-CD4 BV421 (GK1.5), anti-CD8α PerCP-Cy5.5 (53–6.7), anti-CD25 PE (3C7), and anti-TCRβ chain PerCPCy5.5 (H57-597) from BioLegend (San Diego, CA, USA), and anti-CD3ε FITC (145-2C11) from BD Biosciences (San Jose, CA, USA).

Analysis of TCR Vβ usage was performed using 15 FITC-conjugated monoclonal antibodies recognizing the TCR chains Vβ2, 3, 4, 5.1/5.2, 6, 7, 8.1/8.2, 8.3, 9, 10^b^, 11, 12, 13, 14, and 17^a^ TCRs (BD Biosciences, San Jose, CA, USA). The percentage of Vβ^+^ cells was determined in each subset after gating on CD4^+^, CD8^+^, and DN cells.

Data were collected on a Flow Cytometer (FacsCanto II, BD Biosciences, San Jose, CA, USA) and analyzed using FlowJo Software (Tree Star, Ashland, OR, USA). Cell sorting of thymocyte populations was performed on a FACS Aria IIIu (BD Biosciences, San Jose, CA, USA) carried out by the FACS sorting Core Unit of the University Medical Center Hamburg-Eppendorf.

### Thymocyte Sensitivity to Betamethasone

One million thymocytes from 5-weeks-old untreated C57B1/6J mice were cultured in RPMI-1640 (Gibco, CA, USA) in a 96-well round bottom plate (Thermo Fisher Scientific, Germany) and incubated with increasing concentrations of betamethasone (0.1–100 nM) with or without the addition of 1 µg/ml mifepristone (RU486, Sigma-Aldrich). After 16 h at 37°C, cells were harvested, washed with 1 × Annexin V Binding Buffer (Exbio, Czech Republic) and subsequently stained for linage markers (CD3, CD4, CD8, CD25). For detection of dead and apoptotic cells, Dead Cell Stain-Pacific Orange (Invitrogen, CA, USA) and Annexin V FITC (BD Biosciences, San Jose, CA, USA) were used according to the manufacturer’s instructions.

### Autologous Mixed Lymphocyte Reaction (AMLR)

Pooled single-cell suspensions from inguinal, axillary, brachial, lumbar, and superficial cervical lymph nodes were stained with the cell proliferation dye eFluor 670^®^ (eBioscience, San Diego, CA, USA) in order to differentiate proliferating and non-proliferating cells. Shortly, lymph node cells were washed with PBS and resuspended in 2 µM eFluor 670^®^ dye in PBS for 10 min at 37°C in the dark. Labeling was stopped by adding 4–5 volumes of cold complete medium (RPMI 1640, 10% FCS, 1% penicillin/streptomycin, 2 mM l-glutamine, 50 µM 2-Mercaptoethanol) and incubated on ice for 5 min. Labeled cells were then cultured in complete medium in the presence or absence of IL-2 (100 U/ml). Cells were plated at 1 × 10^5^/well in a 96-well plate (Thermo Fisher Scientific, Germany) for up to 5 days at 37°C in 5% CO_2_. After 3, 4, and 5 days of culture, lymph node cells were collected, washed with PBS and stained with Dead Cell Stain-Pacific Orange and anti-TCRβ chain PerCPCy5.5, anti-CD4 APC-eFluor 780, and anti-CD8α PE-Cy7 antibodies. The proliferation of CD4^+^, CD8^+^, and DN cells was evaluated according to eFluor dilution measured by flow cytometry (FacsCanto II, BD Biosciences).

### Disease Scoring

#### MRL/lpr Mice

Organ weights were measured and weight index (mg/g body weight) of lymphoid organs (spleen and lymph nodes) was calculated as follows: organ weight/body weight × 1,000.

##### Measurement of Double-Stranded (ds) DNA Autoantibodies

Blood samples were collected every 2 weeks starting at 6 weeks of age and serum autoantibodies specific for dsDNA were measured by enzyme-linked immunosorbent assay (ELISA). 96-well plates were coated with 5 µg/ml calf thymus dsDNA (Sigma-Aldrich) at 4°C overnight. Plates were blocked for 2 h with 1% BSA in PBS followed by incubation with diluted mouse serum for 2 h at RT. Bound anti-dsDNA autoantibodies were detected with a 1:2,000 diluted sheep anti-mouse IgG-HRP (Amersham, UK) (2 h, RT), and peroxidase reaction was developed using 2,2′-Azino-bis(3-ethylbenzothiazoline-6-sulfonic acid) diammonium salt (Sigma-Aldrich, Germany). Absorbance was measured at 405 nm using a multilabel plate reader (Victor3, PerkinElmer, MA, USA), data were analyzed using Prism software (GraphPad Software, La Jolla, CA, USA).

##### Measurement of Proteinuria

Mice were housed in metabolic cages for urine collection for 2 h every 2 weeks starting at 6 weeks of age. Albuminuria was determined by ELISA (Mouse Albumin ELISA Quantitation Set; Bethyl Laboratories, Inc.).

#### Non-Obese Diabetic (NOD) Mice

Starting at 10 weeks of age, adult mice were monitored daily for urine glucose using Glucocard strips (Menarini, Barcelona, Spain), until 25 weeks of age. Mice with glycosuria were confirmed diabetic when the blood glucose level was >300 mg/dl. The degree of immune cell infiltration in the islets (insulitis score) was determined at the end of the study in all non-diabetic mice. Briefly, pancreata were snap frozen in an isopentane/cold acetone bath. Cryosections of 5 µm were obtained at non-overlapping levels, stained with hematoxylin and eosin (H&E), and analyzed by two independent investigators in a blind fashion. A minimum of 40 islets per animal was analyzed. Insulitis was scored as described elsewhere (31): 0 = no insulitis; 1 = peri-insular inflammation; 2 = infiltration below 25%; 3 = infiltration 25–75%; 4 = infiltration above 75%.

#### EAE Mouse Model

For induction of EAE, C57BL/6J mice were immunized s.c. with 200 µg MOG_35–55_ peptide (Schafer-N, Copenhagen, Denmark) in complete Freund’s adjuvant (Difco Laboratories, LI, USA) containing 4 mg/mL *Mycobacterium tuberculosis* (Difco). In addition, 200 ng pertussis toxin (Calbiochem, San Diego, CA, USA) was injected i.v. on the day of immunization and 48 h later. Animals were scored daily for clinical signs by the following system: 0 = no clinical deficits; 1 = tail weakness; 2 = hind limb paresis; 3 = partial hind limb paralysis; 3.5 = full hind limb paralysis; 4 = full hind limb paralysis and forelimb paresis; and 5 = premorbid or dead. Animals reaching a clinical score ≥ 4 had to be killed according to the regulations of the Animal Welfare Act. Investigators were blinded for prenatal treatment during the experiments.

### Gene Expression Analysis

RNA was extracted from sorted T cell subsets or from thymocytes after *in vivo* or *in vitro* treatment using the RNeasy Mini Plus kit (QIAGEN, Hilden, Germany) and cDNA was synthesized with the M-MLV Reverse Transkriptase kit (Invitrogen). TaqMan gene expression assay (LifeTechnologies, CA, USA) was used to detect *GAPDH* (Hs02758991_g1) expression. 18S and FoxP3 expression were determined using SYBR^®^ green and following primers: 18S forward: 5′-CGGCTACCACATCCAAGGAA-3′ 18S reverse: 5′-GCTGGAATTACCGCGGCT-3′; FoxP3 forward: 5′-GGCCCTTCTCCAGGACAGA-3′ FoxP3 reverse: 5′-GCTGATCATGGCTGGGTTGT-3′.

### Statistics

Statistical analysis of TCR Vβ chain usage was performed with Matlab R2016b (The Mathworks). The fractions of positive cells for each Vβ chain, as well as the remaining fraction of cells that was not positive for any of the measured Vβ chains (other Vβ), were log or square-root transformed to obtain normally distributed data. Using *N*-way ANOVA, the Vβ chain fractions for each cell type were correlated to treatment and possible interaction terms, with correction for litter size and subject nested within treatment. Upon reaching statistical significance, pairwise comparison with Fisher’s least significant difference correction was performed to identify which Vβ chain(s) were differently expressed upon treatment. For calling statistical significance, alpha of 0.05 was applied in all analyses.

Additional statistical analysis, including unpaired Student’s *t*-test, Gehan–Breslow–Wilcoxon test, and Mantel–Cox test were performed with GraphPad Prism Software (La Jolla, CA, USA) and are indicated in the corresponding figure legends. *P < 0.05, **P < 0.01, ***P < 0.001, and ****P < 0.0001.

### Study Approval

This study was carried out in accordance with the recommendations of the Declaration of Helsinki for animal experimental investigation and the Principles of Laboratory Animal Care (NIH pub.85–23 revised 1985). The protocol was approved by the local animal ethics committees (ethical approval 119/13 and 122/12 obtained from the state authority of Hamburg, and DMAH8948 obtained from the Generalitat de Catalunya).

## Results

### Prenatal Glucocorticoid Treatment Results in Apoptosis of DP Thymocytes in the MRL/lpr Offspring

**Figure 1:**
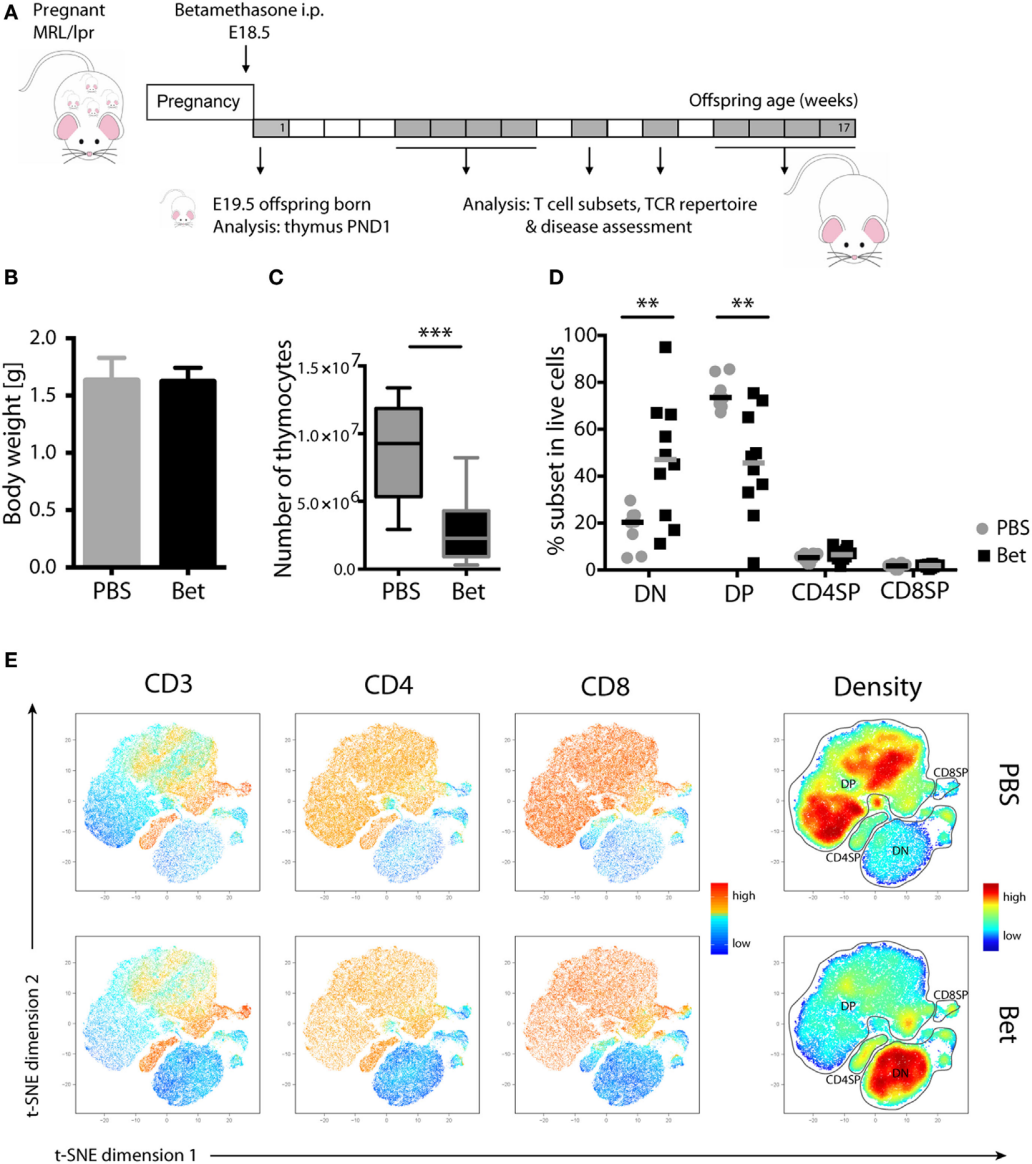
Loss of double-positive (DP) thymocytes in the offspring of MRL/lpr mice after prenatal betamethasone treatment. **(A)** Schematic representation of the MRL/lpr mouse model. **(B)** Body weight from prenatally betamethasone (Bet) and vehicle-treated (PBS) MRL/lpr mice (*n* = 42–44 per group, male and female). **(C)** Total number of thymocytes (*n* = 10–11 per group, male). **(D)** Frequency of CD4^−^CD8^−^ double-negative (DN), CD4^+^CD8^+^ DP, and mature CD4^+^ and CD8^+^ single-positive (SP) thymocytes (*n* = 8–10 per group, male). **(E)** t-SNE representation of the surviving cells in the thymic compartment after prenatal betamethasone treatment according to the expression of CD3, CD4, and CD8. Each dot represents a cell, and the colors show levels of expression of the indicated markers. The plots show one representative animal per group. The rightmost plots display the cell abundance (density) for each population. All analyses were performed at postnatal day 1 (PND1). Unpaired Student’s *t-*test was used for statistical analysis, **P < 0.01 and ***P < 0.001.

We have previously shown in C57BL/6J mice that prenatal betamethasone treatment causes a profound reduction in the thymic volume and cell numbers in the offspring (16). The question that arises is if the massive death and subsequent accelerated replenishment of the thymus has consequences upon T cell repertoire selection and immunity later in life. To explore this, we took advantage of the MRL/MpJ-*Fas^lpr^* (hereafter referred to as MRL/lpr) autoimmunity-prone mouse strain, which spontaneously develops lupus-like glomerulonephritis and vasculitis as result of autoantibody production and immune complex deposition (32). In this strain, we first sought to confirm the effects of prenatal glucocorticoid treatment on the thymus. After treating the pregnant dams (E18.5) with betamethasone (Figure 1A), at postnatal day 1 (PND1) we did not observe any difference in the weight of the pups (Figure 1B), but a drastic reduction in the number of living thymocytes (Figure 1C). Not surprisingly, thymocyte loss was caused by a massive reduction in the CD4^+^CD8^+^ DP compartment and, as a consequence, a compensational increase in the frequency of DN cells (Figures 1D,E) could be observed. This effect was transient, since in the adult offspring the percentage of DP thymocytes was similar in both groups (not shown). Figure 1E shows a direct comparison of the composition of the thymocyte compartment in a sham- (upper row) vs. a betamethasone-treated (lower row) animal. The density plot in the right panels demonstrates the shift from maximal representation of DP cells in the untreated animals to a maximum of DN cells in the animals treated with betamethasone. Importantly, the range of DP cell loss within a litter was highly variable, with some animals displaying marginal effects while others have nearly lost the DP compartment (Figure 1D). This variation is likely the result of different exposure of each individual fetus to betamethasone (16). The frequencies of CD4SP and CD8SP cells remained similar, although we could notice a reduction in absolute cell counts (not shown).

### Prenatal Betamethasone Treatment Leads to Changes in the T Cell Repertoire of MRL/lpr Offspring

**Figure 2:**
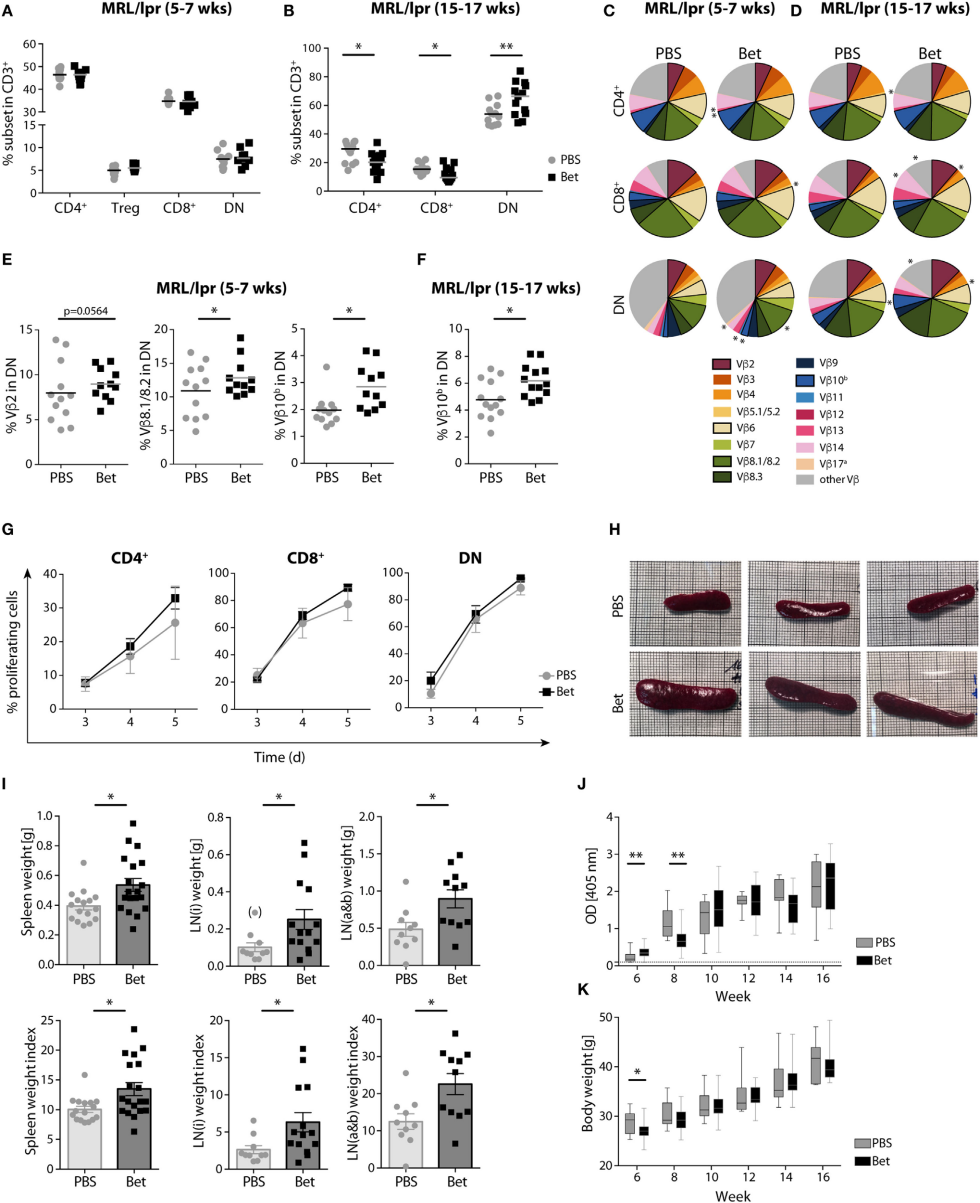
Long-term effects of prenatal steroid treatment in MRL/lpr offspring. Frequencies of spleen T cell subsets CD4^+^, Treg cells, CD8^+^, and DN cells at **(A)** 5–7 weeks and **(B)** 15–17 weeks of age (*n* = 11–13 per group, male and female). **(C,D)** T cell receptor (TCR) Vβ chain usage among CD4^+^, CD8^+^, and DN splenocytes at **(C)** 5–7 (*n* = 11–12 per group, male and female) and **(D)** 15–17 weeks of age (*n* = 13 per group, male and female) was assessed by flow cytometry. Each of the 15 analyzed TCR Vβ chain families is represented by a segment proportional in size to its frequency (which on average cover between 61 and 91% of all cells within the subsets, the segment “other Vβ” indicates Vβ chain expression not covered by our panel of TCR Vβ chain-specific Abs). Segments marked with an asterisk differ statistically significant between the groups (PBS or Bet), framed segments indicate TCR Vβ chains important for autoimmune disease in MRL/lpr mice. **(E)** Detailed results for the relevant TCR Vβ2, Vβ8.1/8.2, and Vβ10^b^ in DN spleen cells at 5–7 weeks of age. **(F)** Detailed results for the relevant TCR Vβ10^b^ in DN spleen cells at 15–17 weeks of age. *N*-way ANOVA was used for statistical analysis of TCR Vβ chain usage. **(G)** Proliferation of autoreactive CD4^+^, CD8^+^, and DN cells from female MRL/lpr mice (*n* = 3–4 per group at 6–8 weeks). Pooled lymph node cells (1 × 10^5^) were cultured in the presence of IL-2 (100 U/ml) without the addition of exogenous TCR stimulation for 3–5 days. Extent of cell proliferation was analyzed by flow cytometry based on eFluor 670 dilution. Values are expressed as mean ± SEM. **(H–K)** Disease parameters in MRL/lpr mice: **(H)** Size of spleen at 15 weeks of age. Effect of prenatal betamethasone treatment on **(I)** spleen (*n* = 16–19 per group) and lymph node (i = inguinal, *n* = 10–14 or a&b = axillary and brachial, *n* = 10–11 per group) weights and indices (mg/g body weight), **(J)** serum levels of dsDNA autoantibodies (*n* = 7–28 per group and time point), and **(K)** body weight (*n* = 9–29 per group and time point). Unpaired Student’s *t-*test was used for statistical analysis; *P < 0.05 and **P < 0.01.

We next asked if the massive perinatal thymocyte death had long lasting consequences on the peripheral T cell compartment. While the MRL strain is prone to autoreactivity, the defect in *fas* (*lpr*, lymphoproliferation) leads to uncontrolled expansion and accumulation of autoreactive cells in the peripheral lymphoid organs, and accelerates, rather than initiates, disease (33). These autoreactive cells harbor the phenotype CD3^+^B220^+^CD4^−^CD8^−^ (DN T cells), and steadily increase with disease progression to become the most abundant subset in spleen and lymph nodes. Importantly, the TCR Vβ usage of public clones in the enlarged lymph nodes and in T cells infiltrating the kidneys of diseased animals is limited to a few families, including Vβ2, Vβ6, Vβ8.2, Vβ8.3, and Vβ10, underlining their pathogenic relevance (34–36). To assess if prenatal glucocorticoid treatment had an effect on the autoimmune repertoire, we performed flow cytometric analysis of TCR Vβ chain usage in peripheral CD4^+^, CD8^+^ and DN T cells of young (5- to 7-week old) and aged (15- to 17-week old) offspring of mothers treated with vehicle or betamethasone. While the frequencies of CD4^+^, CD8^+^, and DN T cells at 5–7 weeks of age, before appearance of disease symptoms, were similar in both groups, accumulation of DN T cells in the older animals was clearly more prominent in the group that had received betamethasone prenatally (Figures 2A,B). Moreover, analysis of Vβ chain usage on the different T cell subsets indicated a bias in TCR receptor Vβ expression in these mice, with higher representation of the potentially autoreactive Vβ2- (7.97 vs 8.95%, *P* = 0.0564), Vβ8.1/8.2- (10.91 vs 12.85%, *P* = 0.0379), and Vβ10^b^- (1.97 vs 2.85%, *P* = 0.0113) expressing DN T-cells in young animals (5–7 weeks) (Figures 2C,E) and further differences in the frequency of Vβ5.1 CD4^+^ and CD8^+^ cells, Vβ11 in CD4^+^ T cells, and Vβ12 in CD4^+^ T and DN T cells (Table S1 in Supplementary Material). Similarly, the TCR Vβ usage in MRL/lpr animals with advanced disease (at 15–17 weeks of age) was also biased, with differences in the frequency of Vβ14 (7.46 vs 8.46%, *P* = 0.0114) CD8^+^ T cells and, as in young animals, in the disease-relevant Vβ10^b^ (4.78 vs 6.18%, *P* = 0.0389) bearing DN cells (Figures 2D,F; Table S2 in Supplementary Material).

We next sought to determine whether the changes observed in the TCR Vβ repertoire resulted in a higher proliferation, which would reflect the presence of more autoreactive cells. For this, we performed AMLR to measure proliferation of autoreactive T cells in response to endogenous antigens. While no external TCR stimulus was given, IL-2 was added to the cultures to support the incipient proliferation of the few autoreactive precursors. Although not significant, our data show higher proliferation rates of both CD4^+^ and CD8^+^ cells in the betamethasone-exposed animals (Figure 2G). As expected, DN cells showed maximal proliferation, and no differences were found between the treated and non-treated groups, since the accumulating DN cells are chronically activated in both groups.

The *lpr* defect in this mouse strain leads to a progressive enlargement of the lymphoid organs, enhancing the disease phenotype of the MRL strain (33). Therefore, we would expect that a T cell repertoire biased toward more autoreactivity would result in larger lymphoid organs. In agreement with increased amounts of pathogenic TCR Vβ families and enhanced T cell proliferation, the spleens and lymph nodes were considerably larger in the animals whose mothers had been treated with betamethasone (Figures 2H,I), supporting the concept of a more autoreactive T cell repertoire. However, a higher frequency of autoreactive T cells and enlarged lymphoid organs did not translate in accelerated course of disease, as assessed by the levels of dsDNA autoantibodies (Figure 2J) and proteinuria (Figure S1 in Supplementary Material) from 6 to 16 weeks of age, which were similar in both groups. Of note, prenatal betamethasone treatment resulted in a transiently lower body weight (Figure 2K) and higher production of ds-DNA specific IgG autoantibodies (Figure 2J) at 6 weeks of age, but this difference disappeared soon after. Thus, prenatal treatment of the pregnant females with betamethasone elicits drastic thymocyte death in the postnatal thymus, which results in persistent changes in the TCR repertoire promoting the expansion of autoreactive Vβ families.

### Prenatal Betamethasone Protects from T1D Development in NOD Mice

**Figure 3:**
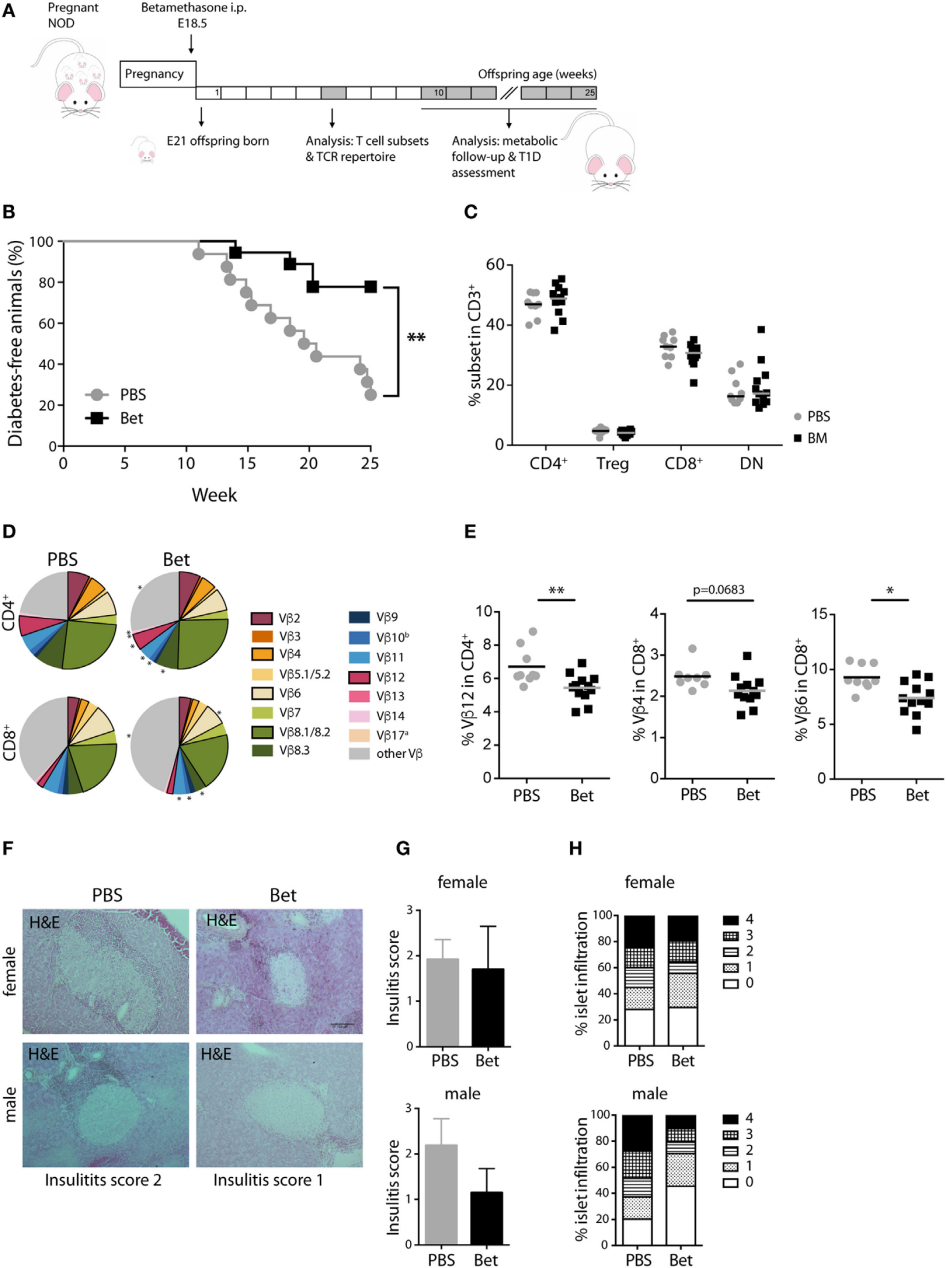
Prenatal steroid treatment reduces insulitis and the incidence of T1D in NOD mice. **(A)** Schematic representation of the NOD mouse model. **(B)** Percentage of T1D-free NOD mice during 25 weeks of follow-up (*n* = 16–18 females per group). Gehan–Breslow–Wilcoxon test was used for statistical analysis. **(C)** Frequency of CD4^+^, Treg cells, CD8^+^, and DN splenocytes from female NOD mice at 6 weeks (*n* = 9–12 per group). **(D)** Comparison of Vβ chain usage between NOD offspring from betamethasone- (Bet) and sham-treated (PBS) dams at 6 weeks of age (*n* = 9–12 per group, female). Each T cell receptor (TCR) Vβ chain among CD4^+^ and CD8^+^ T cells is represented by a segment proportional in size to its frequency (which on average cover between 55 and 78% of all cells within the T cell subsets, the segment “other Vβ” indicates Vβ chain expression not covered by our panel of TCR Vβ chain-specific Abs). Segments marked with a star differ statistically significant between the groups (PBS or Bet), framed segments indicate TCR Vβ chains important for autoimmune disease in NOD mice. **(E)** Detailed results for the relevant TCR Vβ12 in CD4^+^ and TCR Vβ4 and Vβ6 in CD8^+^ spleen cells. *N*-way ANOVA was used for statistical analysis of TCR Vβ chain usage. **(F)** Representative H&E stained sections of pancreata from male and female T1D-free mice at sacrifice (25 weeks). **(G)** Insulitis score and **(H)** percentage of islets (presented as degree of infiltration: 0 = no insulitis, 1 = peri-insular inflammation, 2 = infiltration < 25%, 3 = infiltration 25–75%, 4 = infiltration > 75%) from non-diabetic male and female (*n* = 3 per group) NOD mice. Unpaired Student’s *t-*test was used for statistical analysis, *P < 0.05 and **P < 0.01.

Despite the critical role of T cells in the initiation of autoimmunity in MRL/lpr mice, other mechanisms such as the production of pathogenic autoantibodies and renal immune-complex deposition leading to complement activation, contribute fundamentally to disease progression. Therefore, we turned to a second model of autoimmunity with a more direct involvement of T cells in the immune pathogenesis, the NOD mouse model for T1D to assess persisting effects of prenatal betamethasone treatment. This spontaneous model of autoimmune diabetes is characterized by the activation of autoreactive T cells and the destruction of insulin producing beta-cells in the pancreas. After prenatal administration of vehicle or betamethasone to non-diabetic females (E 18.5), glucose levels were measured in the female offspring for the occurrence of T1D until 25 weeks of age (Figure 3A). Surprisingly, we found that fetal glucocorticoid exposure was protective against the development of T1D in females (Figure 3B). At 25 weeks of age, only 25% of the NOD females in the control group were disease-free, while in the betamethasone treatment group it was 78%. Moreover, T1D onset was delayed in the treated group (from 14 weeks of age) when compared to control mice (from 11 weeks of age), consistently with the protective effect of the drug. There were no differences between the two groups in the frequencies of CD4^+^, Treg cells, CD8^+^, or CD3^+^ DN spleen cells at 6 weeks of age (Figure 3C). We next investigated whether the different incidence of disease could be traced to changes in the TCR Vβ repertoire. In the NOD mice, the most commonly found TCR Vβ chains in T cells infiltrating the pancreas are Vβ4 (37), and Vβ12 (38, 39), followed by Vβ8.1, Vβ2, and Vβ6 (40, 41). Our TCR Vβ usage analysis revealed that Vβ12 (6.71 vs. 5.45%, *P* = 0.0077) CD4^+^ T cells, and Vβ4 (2.66 vs. 2.14%, *P* = 0.0683) and Vβ6 (9.28 vs. 7.39%, *P* = 0.0124) CD8^+^ T cells were underrepresented in the spleen of the female NOD mice whose mothers received betamethasone (Figures 3D,E; Table S3 in Supplementary Material). This lower frequency of pathogenic TCRs was reflected in the lower degree of immune cell infiltration in the pancreas of animals that had not yet developed T1D at 25 weeks of age, determined in a second mouse cohort (Figures 3F–H). Here, the incidence was somewhat lower in both groups, but still 46% of sham-treated females were disease-free at 25 weeks, while 62% of the betamethasone-treated had normal glucose levels (*P* = 0.0847, Figure S2 in Supplementary Material). Therefore, we conclude that a bias in the T cell repertoire caused by prenatal glucocorticoid treatment contributes to protection from T1D in the NOD mice.

### Prenatal Betamethasone Does Not Induce Long Lasting Changes in the Treg Cell Compartment

**Figure 4:**
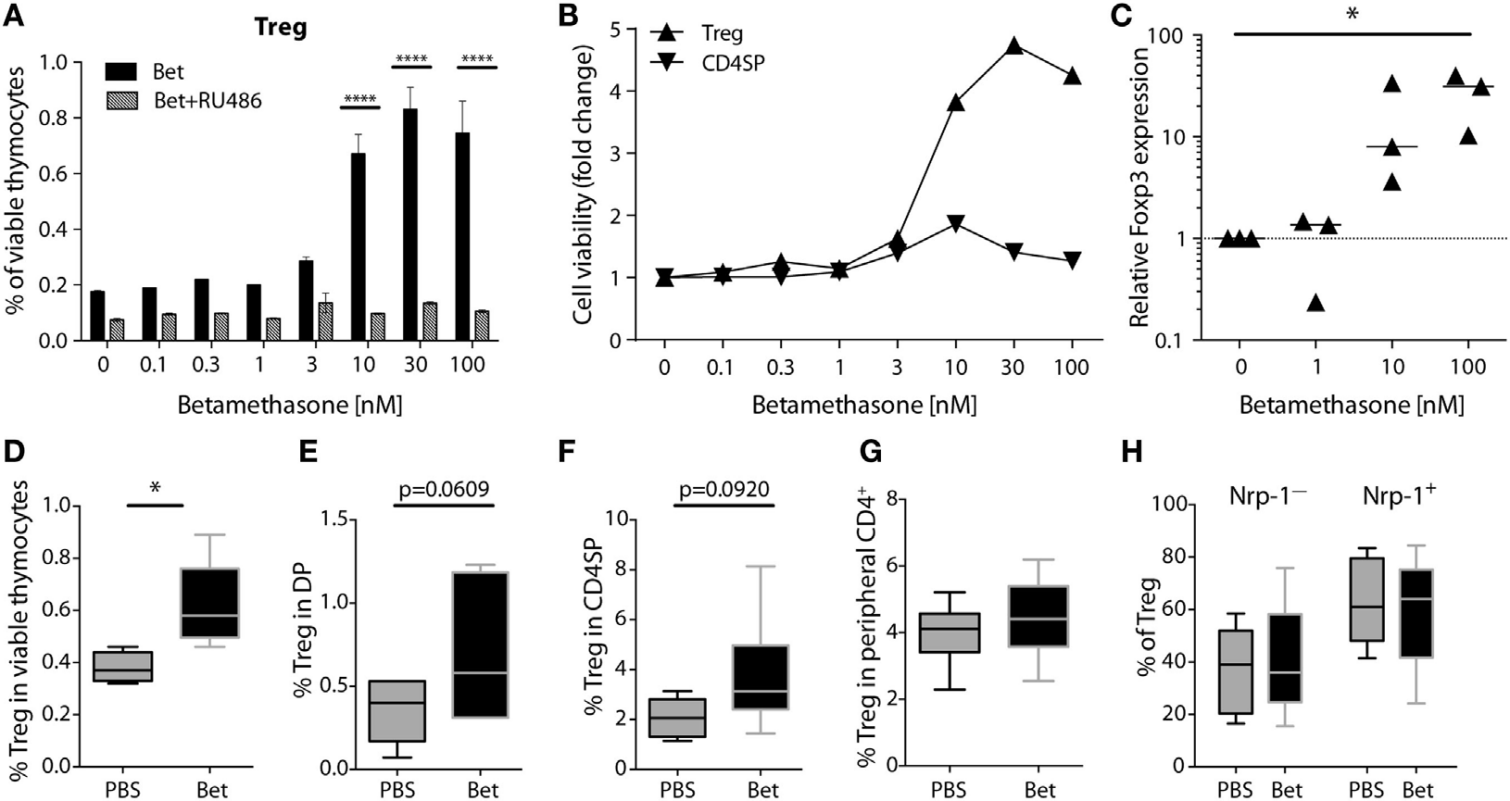
Treg precursor cells are less sensitive to betamethasone than conventional CD4^+^ cells. **(A)** Percentage of Treg cells after *in vitro* treatment of thymocytes (C57BL/6J) with increasing concentrations of betamethasone (0.1–100 nM) in the presence or absence of mifepristone (RU486) for 16 h. **(B)** Cell viability of Treg cells and conventional CD4 single-positive T cells is displayed as fold change in comparison to the negative control (no betamethasone). **(C)** Expression levels of Foxp3 mRNA in thymocytes after *in vitro* treatment with betamethasone (0–100 nM) for 16 h. Untreated thymocytes were used as calibrator. **(D)** Frequency of surviving Treg cells in thymocytes of 5-week-old female C57BL/6J mice (*n* = 5 per group) 24 h after injection of 0.1 mg betamethasone or PBS. **(E)** Frequency of Treg precursor cells in double-positive or **(F)** Treg cells in CD4SP thymocytes from the offspring of prenatally treated dams (*n* = 5–7 per group) at postnatal day 0 (PND0). **(G)** Frequency of Treg (*n* = 15 per group), and **(H)** frequency of Neuropilin-1 positive (tTreg cells) and negative (pTreg cells) cells (*n* = 7–11 per group) in splenocytes at 5–7 weeks of age after prenatal betamethasone or vehicle treatment. Unpaired Student’s *t-*test was used for statistical analysis, *P < 0.05, **P < 0.01, and ****P < 0.0001.

In addition to changes in TCR Vβ usage, another possibility that could affect development of autoimmunity is the quantity or quality of Treg cells. Treg cells are less sensitive to glucocorticoid challenge than conventional CD4^+^ cells (42, 43). However, it is not known if prenatal treatment with betamethasone results in enrichment of Treg cells in the newborn and, more importantly, if there are consequences for the peripheral Treg cell compartment later in life. In a first set of experiments we exposed thymocytes from 6 weeks old (not prenatally treated) C57BL/6J mice to increasing concentrations (0.1–100 nM) of betamethasone *in vitro* and measured the survival of Treg cells compared to conventional CD4^+^ T cells. We used mifepristone (RU486), a synthetic steroid with potent anti-glucocorticoid properties, to confirm the specificity of the betamethasone effects. We found that the relative frequency of surviving CD4^+^CD25^+^ SP thymocytes (which in the naïve mice are considered Treg cells) increased in response to betamethasone in a dose-dependent manner, and this effect was completely inhibited by the addition of RU486 (Figure 4A). More than a threefold increase in the frequency of Treg cells compared to the untreated controls was achieved at 10 nM (Figure 4A). In contrast, conventional CD4^+^ single-positive thymocytes did not significantly change in proportion with increasing concentrations of betamethasone (Figure 4B). To confirm that it is indeed the Treg cell subset the one increasing, we performed RT-PCR on the cultured cells and found increased Foxp3 expression at higher betamethasone concentration (Figure 4C). We obtained similar results when thymocytes derived from untreated Foxp3^RFP^ reporter mice were treated with betamethasone and Foxp3 expression could be monitored by flow cytometry (Figure S3 in Supplementary Material). To test the effects of glucocorticoids on the frequency of Treg cells *in vivo*, we treated 6 weeks old mice i.p. with 0.1 mg betamethasone. Analysis of the thymus 24 h post-treatment revealed a net increase in CD4^+^CD25^+^ in the surviving cells (Figure 4D). Finally, to find out if prenatal betamethasone exposure has an effect on the frequency of neonatal Treg cells, we treated pregnant females and analyzed Treg precursor cells in the DP thymocytes and in the CD4SP subpopulations in the offspring at postnatal day 0. As shown in Figure 4E, we could detect an increased frequency of DP CD25^+^ Treg precursor cells and a slightly elevated proportion of CD4^+^CD25^+^ thymic-derived Treg cells 24 h post-treatment (Figure 4F). This neonatal bias, however, did not persist until adulthood, since the percentage of Treg cells in the spleen of animals at 5–7 weeks of age was similar in both groups (Figure 4G). Moreover, no differences were observed in the expression of neuropilin-1 (Figure 4H) or CD62L (not shown), indicating similar percentages of thymic and peripherally induced Treg cells (44), and similar levels of activation in both groups. Proliferation and inflammatory cytokine production of spleen T cells at this age was also comparable in both groups (not shown). Our data demonstrate that Treg thymocyte precursor cells are less sensitive than conventional CD4^+^ precursors to prenatal administration of betamethasone, resulting in a transient increase in the frequency of Treg precursor cells after birth. However, these differences are not persisting, and do not elicit changes in the future Treg cell compartment.

### Prenatal Betamethasone Has No Effect on Disease in the Context of a Highly Restricted Pathogenic Repertoire

**Figure 5:**
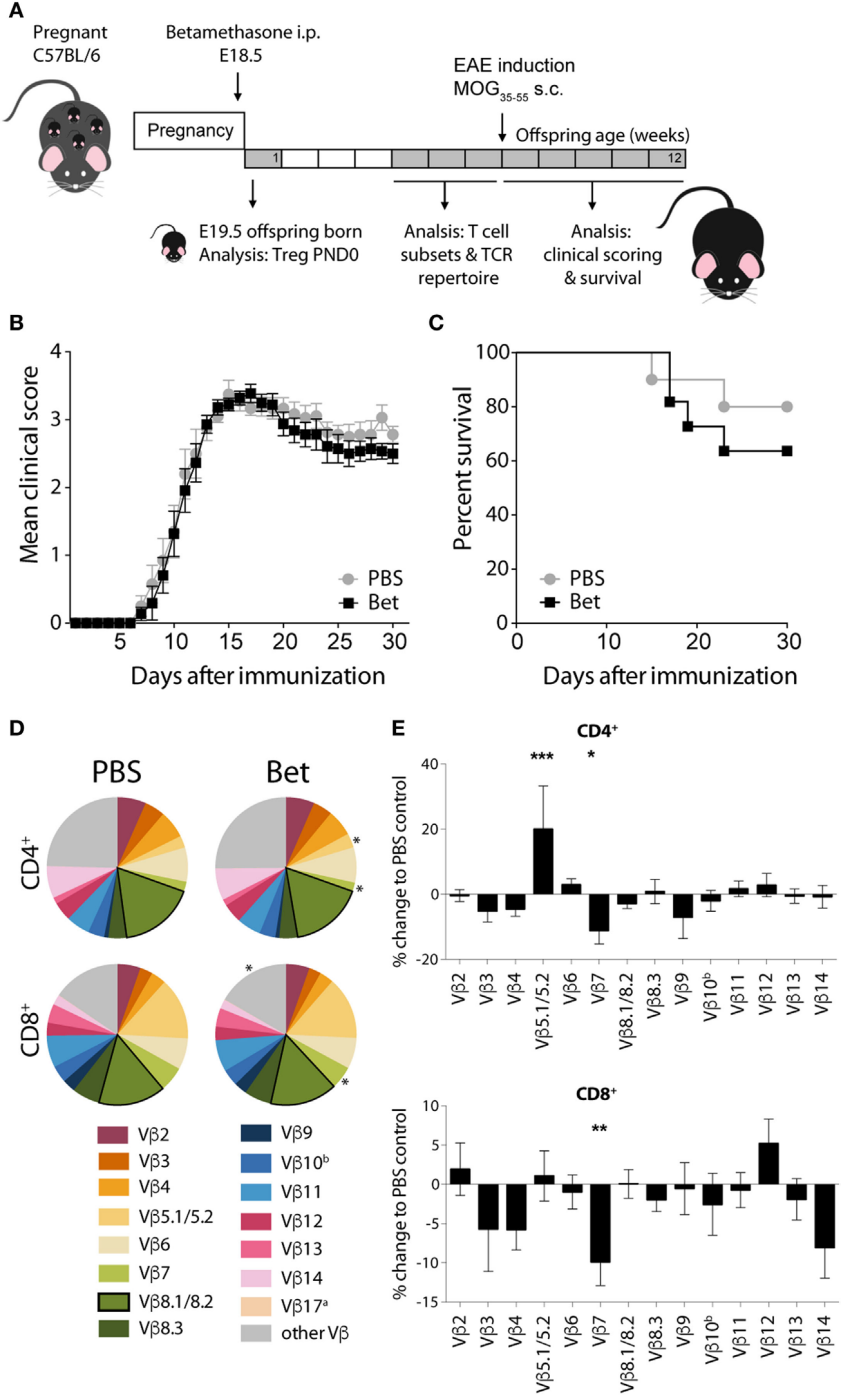
Prenatal betamethasone does not alter the onset or the course of experimental autoimmune encephalomyelitis (EAE) despite changes in the T cell receptor (TCR) Vβ repertoire. **(A)** Schematic representation of the EAE mouse model. **(B)** EAE clinical scores and **(C)** survival of C57BL/6J mice (*n* = 9–11 per group, male and female) after MOG_35–55_ EAE induction in offspring of dams prenatally treated with betamethasone or PBS. Statistical analysis of survival was performed with Mantel–Cox test. **(D)** Comparison of Vβ chain usage between C57BL/6J offspring from betamethasone- or sham-treated mothers. Each TCR Vβ chain among CD4^+^ and CD8^+^ splenocytes is represented by a segment proportional in size to its frequency (which on average cover between 74 and 84% of all cells within the T cell subsets, the segment “other Vβ” indicates Vβ chain expression not covered by our panel of TCR Vβ chain-specific Abs). Segments marked with a star differ statistically significant between the groups (PBS or Bet), framed segments indicate TCR Vβ chains important for autoimmune disease in EAE mice. Data obtained at 5–7 weeks (*n* = 15 per group, male and female). **(E)** TCR Vβ chain usage represented as percent of change to negative control (PBS) ± SEM. *N*-way ANOVA was used for statistical analysis of TCR Vβ chain usage, *P < 0.05, **P < 0.01, and ***P < 0.001.

In the two models of autoimmune disease that we have analyzed, disease develops spontaneously, and the TCR repertoire of the pathogenic cells is oligoclonal. In both cases, we have shown that antenatal betamethasone treatment elicits changes in the TCR Vβ usage affecting the autoreactive T cell repertoire. To address if indeed glucocorticoid-induced changes in the TCR repertoire are responsible for this effect, we took advantage of a model in which only few TCRs are engaged in the pathogenic response, namely EAE induced by MOG_35–55_ peptide. In contrast to the more polyclonal response in the lupus-prone MRL/lpr mouse and in the NOD mouse model, in this induced model of autoimmunity, half of the CNS infiltrating cells bear Vβ8.1 or Vβ8.2 (45–47). We therefore treated pregnant C57BL/6J females with betamethasone at E18.5, and let the offspring develop normally, until injection of the MOG peptide at 6 weeks of age (Figure 5A). After disease induction, mice were examined daily and scored for clinical symptoms of disease for 30 days. No differences could be seen in disease onset, clinical score or survival between the animals whose mothers had been treated with betamethasone or with vehicle (Figures 5B,C). Analysis of the TCR repertoire revealed significant changes in Vβ chain usage between the two groups, namely affecting Vβ5.1/5.2 (2.73 vs. 3.27%, *P* = 0.0004) and Vβ7 (2.24 vs. 1.99%, *P* = 0.0315) in CD4^+^ and Vβ7 (5.79 vs. 5.22%, *P* = 0.0040) in CD8^+^ T cells. However, the frequency of the disease-relevant Vβ8.1/8.2 chains remained unchanged (Figures 5D,E; Table S4 in Supplementary Material). Our data show that prenatal betamethasone is not altering the highly restricted pathogenic TCR repertoire in EAE, and—consequently—the disease course is similar in both groups of mice.

## Discussion

Glucocorticoids are administered to pregnant women at risk of preterm delivery. A single course of betamethasone reduces morbidity related to acute respiratory distress in the neonate, without apparent short-term side effects (6). However, a complete absence of a thymic shadow in X-ray images has been reported for all newborns that received prenatal dexamethasone treatment, while in the majority of healthy gestation-week matched untreated controls the thymus was visible (48). This condition is transient, since control X-rays over the first 4 weeks of life showed a progressive enlargement of the thymic gland (48). To explore possible long-term consequences of prenatal glucocorticoids in humans is a challenge, since women are treated upon symptoms of pre-term birth, which may happen at different time points of pregnancy, and the time span between treatment and birth can also be highly variable. It is therefore not surprising that analysis of cord blood parameters has not yielded conclusive results [reviewed in Ref. (49)]. Interestingly, epidemiological studies in much larger cohorts indicate an increased risk for infection in the neonate (50), and for asthma (51) and T1D (52) in young children that received prenatal steroid treatment.

For the reasons mentioned above in relation to human studies, we decided to use mouse models to explore possible long-term consequences of prenatal steroids upon the immune system. One dose of betamethasone equivalent to what is given in human pregnancy was injected to the dams before delivery. This dose is the minimum dose that showed improved lung maturation compared to vehicle in mice (53). Our experiments showed a drastic reduction in thymic size and cell numbers after prenatal betamethasone in the three mouse strains tested. The thymus plays an essential role for the development of a functional and self-tolerant TCR repertoire. Positive and negative selection events based on TCR avidity for self pMHC, ensure the elimination of non-functional or autoreactive T cells. Glucocorticoids have been shown to regulate the avidity threshold between positive and negative selection, and altered glucocorticoid signaling results in a biased T cell repertoire (26–28, 54, 55). Thus, it is plausible that an excess of glucocorticoids in a moment of active immune development, like the perinatal period, causes changes in the T cell repertoire influencing development of autoimmunity.

Autoreactive T cell clones bearing distinct TCR Vβ chains expand in the target organs of autoimmune disease. For instance, T cells of the Vβ8.2 family recognizing MOG_35–55_ are predominant in the brain of C57BL/6 mice (47), and the repertoire of T cells infiltrating the pancreas of the NOD mouse is skewed to a limited set of families, including Vβ2, Vβ4, Vβ6, Vβ8.1, and Vβ12 (37–41, 56). Interestingly, injection of dexamethasone in the first 3 days of life has been shown to exert long-lasting effects on the expression of several Vβ genes in rats (57). We thus sought to assess the effect of prenatal corticosteroid treatment on the TCR Vβ repertoire in two spontaneous mouse models of autoimmunity. We decided to use flow cytometry to assess TCR Vβ family usage because it allows efficient measurement on the different T cell subsets in individual mice simultaneously, without previous cell sorting. Exploiting this strategy, we observed that Vβ chains that are relevant for the development of T1D in the NOD mouse are underrepresented in the offspring of mothers that were treated with betamethasone. For instance, we found decreased frequencies of peripheral T cells bearing the Vβ4 in mice whose mothers received betamethasone. Vβ4 is utilized by all NOD mice in response to a dominant epitope on glutamic acid decarboxylase 65, and T cell responses to this antigen are already detected in the spleens of young pre-diabetic NOD mice (37), suggesting that this is an important epitope of the primary response. The oligoclonal pattern of islet infiltrating T cells in the NOD model in pre-diabetic mice (40, 41) is a strong indication of local antigen-driven expansion of a limited number of clonotypes, at least at the start of the disease, before epitope spreading. Thus, even subtle changes in the composition of the TCR repertoire, if affecting the relevant specificities, are likely to influence disease development. Not surprisingly, disease severity remained unchanged after prenatal betamethasone treatment in MOG-induced EAE, where T cells from the TCR Vβ8.1/8.2 families, which account for half of the cells infiltrating the central nervous system (46), were not affected by prenatal steroid treatment.

In contrast to the NOD model, we found that Vβ chains known to be expanded in the lymph nodes of MRL/lpr mice are more prominent in the offspring of betamethasone-treated mothers. In this particular mouse strain, the defect in *Fas^lpr^* gene promotes the accumulation of activated autoreactive cells with the CD3^+^CD4^−^CD8^−^ DN phenotype in peripheral lymphoid organs (33, 58). The expanded population of the abnormally proliferating lymph node T cells in MRL/lpr mice is heterogeneous, but Vβ gene expression is skewed toward members of the Vβ2 and Vβ8 families in the DN population (35, 36, 59, 60). The pathogenic nature of Vβ8-bearing T cells is underpinned by the lack of disease in animals that have been treated with anti-TCR Vβ8 antibodies (61). Our data show that Vβ2, Vβ8.1/8.2 and Vβ10^b^ are overrepresented in the DN population in the offspring of betamethasone-treated mothers, indicating a larger contingent of autoreactive cells in these animals. This is further supported by the higher proliferation of CD4^+^ and CD8^+^ T cells in the AMLR, and the larger size of lymphoid organs. Still, we did not observe more severe disease in the treated animals, which is probably due to the fact that although autoreactive T cells contribute to the initiation of disease, the underlying causes for the pathogenesis in the MRL/lpr lupus model lie in the activation of complement by immune complex deposition in the kidney and blood vessels, and inflammatory responses triggered by defects in the clearance of dying cells (62, 63).

How do prenatal glucocorticoids exert their effect on the T cell repertoire? Using different strategies of glucocorticoid-receptor deficiency it has already been shown that diminished glucocorticoid signaling can alter the positive and negative selection avidity thresholds, or create “holes” in the TCR repertoire (26–28). Indeed, lymph nodes and spleen were smaller and the TCR repertoire was less autoreactive in MRL/lpr animals with deficient glucocorticoid receptor expression in thymocytes (26), which is the opposite effect of what we have observed after prenatal excess of glucocorticoids. Mechanistically, this could be due to the ability of glucocorticoids to regulate T cell activation-related processes, thus influencing the outcome of thymic selection. Another possibility is that glucocorticoids alter the TCR repertoire by modulating the transcription of endogenous retroviruses encoding for superantigens (64). Steroid hormones stimulate the expression of HERV-K in human cell lines (65), and a recent study has shown that all endogenous retroviral promoters tested responded to dexamethasone treatment with changes in activity in more than 20 mouse strains (66). Since in the process of developing tolerance to superantigens the TCR-reactive Vβ chains are partially or completely deleted in the thymus (67), glucocorticoid-induced changes in the transcription of endogenous retroviruses could be the underlying cause for the differences that we find in the peripheral TCR repertoire. Indeed, endogenous retroviral sequences induced autoreactive T cells when injected into neonate NOD, but not C57BL/6 mice (68), highlighting the importance of this pathway for the modulation of the T cell repertoire, but also the strain-specific effects (66, 69). In humans, reactivation of endogenous retroviruses has been associated to several autoimmune diseases, including T1D, multiple sclerosis and systemic lupus erythematosus [reviewed in Ref. (70)]. For instance, viral particles of the human endogenous retrovirus HERV-K have been isolated from pancreatic islets of T1D patients, suggesting a possible role in the disease (71). HERV-K encodes for a superantigen that can efficiently stimulate Vβ7 CD4^+^ T cells, which are expanded in the pancreas of patients with T1D (72, 73). Notably, TCR Vβ7 CD4^+^ precursors were deleted by HERV-K18 in an *in vitro* model of human negative selection (74), altogether linking glucocorticoid-induced transcription of endogenous retroviruses, TCR Vβ repertoire selection and autoimmunity also in the human system.

The observed disease protection in the NOD model is intriguing. Obviously, prenatal exposure to glucocorticoids can alter the epigenome of the offspring, affecting multiple organs and pathways (75). Therefore, in addition to the observed effects on TCR repertoire, we cannot discard epigenetic effects on glucose metabolism or pancreatic beta cell development as disease modifiers (76), and research on this topic is ongoing. Moreover, changes in the microbiome are likely to influence immune system development in the offspring (77). In this context, prenatal dexamethasone was found to induce epigenetic changes affecting genes involved in intestinal barrier function, subsequently influencing bacterial colonization at two weeks after birth (78). Interestingly, changes in the intestinal microbiota or a diet rich in the microbial metabolites acetate and butyrate have a profound influence on the development of T1D in the NOD mouse by decreasing the frequency of autoreactive T cells or boosting regulatory function (79, 80).

Foxp3^+^ Treg cells constitute another plausible player in disease protection. Naturally occurring Treg cells develop in the thymus, originating from thymocytes with high affinity for self pMHC complexes (81), and are also subjected to the effects of prenatal glucocorticoids. In line with published data (42), our results obtained from different *in vitro* and *in vivo* settings show that Treg precursor cells are less sensitive to glucocorticoid-induced cell death than their non-Treg cell counterparts, and this has also been shown for mature Treg cells (43). This could be due to several reasons, including an enhancer effect of glucocorticoid signaling on *foxp3* gene expression (82, 83), or Treg cell generation driven by intrathymic TGF-β production upon thymocyte apoptosis (84). Thus, either by preventing apoptosis of Treg cells or by directly inducing Treg cell differentiation, glucocorticoids support enhanced Treg cell development, noticeable shortly after birth. This effect, however, does not persist into adulthood, since we do not see any difference in Treg cell frequencies or function (not shown). In line with our data, adult GR-deficient animals had normal frequencies of Treg cells in the periphery (28). A particular population of Treg cells generated perinatally are crucial for the maintenance of tolerance to self-antigens, and the presence of these cells prevented insulitis in the NOD mice (22). We do not favor the notion that the perinatally generated Treg cells are responsible for protection in our model, because if prenatal steroids would specifically enhance development of this highly protective population, their influence would be noticeable not only in the NOD model, but also in the EAE model, which is susceptible to even small variations in the Treg cell compartment (43). Altogether, we conclude that it is the changes in the TCR repertoire and not the effect on Treg cell development what influences disease susceptibility in the NOD model. Interestingly, a recent study showed a beta cell antigen-specific pro-inflammatory T-helper cell signature in 6-month-old children who years later developed T1D, suggesting early T cell priming in the perinatal period (85).

A limitation of our prenatal betamethasone treatment model is that not all fetuses are equally affected by prenatal steroid treatment [Figure 1D (16)]. At the time point PND1, the relative frequencies of CD4^+^CD8^+^ thymocytes or of granulocytes in spleen can be taken as a proxy for the amount of betamethasone that the fetus received. At 6 weeks of life, however, it is not possible to identify the animals that were only minimally affected by prenatal steroids, and therefore they are also included in the analysis. In this regard, we believe that the long-term effects of prenatal betamethasone might be more prominent than what our results show, since they include animals that were very little affected, and this will not be the case in human singleton pregnancies. A possibility to overcome this problem in the mouse model could be to inject betamethasone postnatally, however, the influence of the incoming microbiome will probably mask the effects of betamethasone. Finally, while our data clearly demonstrate that changes in the TCR repertoire alter the course of autoimmunity, additional T cell-independent mechanisms influencing the outcome of disease are not ruled out.

In this study, we asked whether prenatal exposure to glucocorticoids influences the development of autoimmunity later in life. By analyzing TCR Vβ usage in two mouse strains that spontaneously develop autoimmunity and in the MOG-EAE model, we provide evidence that prenatal betamethasone treatment, by changing the peripheral T cell repertoire, modifies the autoreactive T cell pool, and this may lead to unpredictable variations in the course of autoimmune disease, depending on which T cell clonotypes are affected. Thus, in spite of the undisputable benefits of prenatal glucocorticoids for infants born prematurely, this treatment may be associated with long-term effects on immunity, and it should be used with caution in cases where the risk of preterm birth is only marginal.

## Ethics Statement

This study was carried out in accordance with the recommendations of the Declaration of Helsinki for animal experimental investigation and the Principles of Laboratory Animal Care (NIH pub.85–23 revised 1985). The protocol was approved by the local animal ethics committees (ethical approval 119/13 and 122/12 obtained from the state authority of Hamburg, and DMAH8948 obtained from the Generalitat de Catalunya).

## Author Contributions

AG, CG, ID, and ET designed the experiments. AG, CG, DP-B, JE, ID, NK, and SK performed experiments and acquired data. AG, CG, DP-B, JE, and ID analyzed data and interpreted results. AG and SJ performed statistical analyses. LG conducted data visualization. AG and LG prepared the final figures. H-WM, MF, and MV made intellectual contributions. AG and ET wrote the manuscript. All authors read the manuscript and approved submission.

## Conflict of Interest Statement

The authors declare that the research was conducted in the absence of any commercial or financial relationships that could be construed as a potential conflict of interest.
