# Supplementary material for: Prenatal Administration of Betamethasone Causes Changes in the T Cell Receptor Repertoire Influencing Development of Autoimmunity
Source: Front Immunol. 2017 Nov 13;8:1505. doi: 10.3389/fimmu.2017.01505 (PMC5693859; doi:10.3389/fimmu.2017.01505)
Supplement: Supplementary file 5 [file Data_Sheet_1.PDF]

Supplementary Material

Prenatal Administration of Betamethasone Causes Changes in the T Cell Receptor Repertoire Influencing Development of Autoimmunity

Anna Gieras*, Christina Gehbauer, David Perna-Barrull, Jan Broder Engler, Ines Diepenbruck, Laura Glau, Simon A. Joosse, Nora Kersten, Stefanie Klinge, Hans-Willi Mittrücker, Manuel A. Friese, Marta Vives-Pi, Eva Tolosa

*** Correspondence:** Anna Gieras: a.gieras@uke.de

1. Supplementary Data

Supplementary Figure S1 | Proteinuria in MRL/lpr mice
Supplementary Figure S2 | Prenatal steroid treatment reduces insulitis and the incidence of diabetes in NOD mice
Supplementary Figure S3 | Increased percentage of Foxp3 expressing cells after betamethasone treatment
Supplementary Table S1 |TCR Vβ usage in MRL/lpr mice (5-7 wks) (xlsx file)
Supplementary Table S2 |TCR Vβ usage in MRL/lpr mice (15-17 wks) (xlsx file)
Supplementary Table S3 |TCR Vβ usage in NOD mice (6 wks) (xlsx file)
Supplementary Table S4 |TCR Vβ usage in C57BL/6J mice (5-7 wks) (xlsx file)

2. Supplementary Figures

Supplementary Figure S1

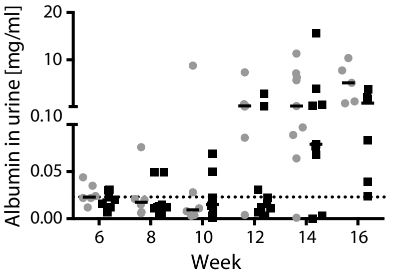

Supplementary Figure S1 | Proteinuria in MRL/lpr mice. Effect of prenatal betamethasone treatment on proteinuria measured by ELISA at indicated time points (n=5-10 per group at 6-16 weeks, female). Student's *t*-test was used for statistical analysis.

Supplementary Figure S2

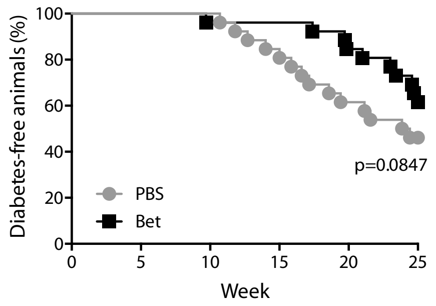

Supplementary Figure S2 | Prenatal steroid treatment reduces insulitis and the incidence of diabetes in NOD mice. Percentage of T1D-free NOD mice during 25 weeks of follow-up (n=26 females per group). Gehan-Breslow-Wilcoxon test was used for statistical analysis.

Supplementary Figure S3

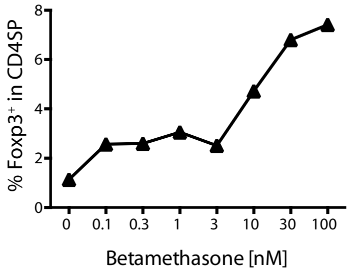

Supplementary Figure S3 | Increased percentage of Foxp3 expressing cells after betamethasone treatment. Frequency of Foxp3-expressing thymocytes in the CD4SP compartment of $Foxp3^{mRFP}$ reporter mice after culture with betamethasone (0.1-100 nM).
